# Microbiological Profiles of Patients with Acute Periprosthetic Joint Infection Undergoing Debridement, Antibiotics, Irrigation and Implant Retention (DAIR)

**DOI:** 10.3390/antibiotics14090873

**Published:** 2025-08-30

**Authors:** Alberto Alfieri Zellner, Niclas Watzlawik, Jonas Roos, Gunnar Thorben Rembert Hischebeth, Ernst Molitor, Alexander Franz, Frank Sebastian Fröschen

**Affiliations:** 1 Department of Orthopaedics and Trauma Surgery, University Hospital Bonn, 53127 Bonn, Germany; alberto.zellner@ukbonn.de (A.A.Z.); watzlawik.n@t-online.de (N.W.); jonas.roos@ukbonn.de (J.R.); alexander.franz@ukbonn.de (A.F.); 2 Institute of Medical Microbiology, Immunology and Parasitology, University Hospital Bonn, 53127 Bonn, Germany; hischebeth@microbiology-bonn.de (G.T.R.H.); molitor@uni-bonn.de (E.M.); 3 Department of Trauma and Orthopedic Surgery, BG Klinik Ludwigshafen, 67071 Ludwigshafen, Germany

**Keywords:** PJI, DAIR, microbiology, revision arthroplasty, DTT, acute periprosthetic infection, microbiological profiles, antibiotic treatment

## Abstract

**Background**: Periprosthetic joint infection (PJI) is one of the most serious complications following total joint arthroplasty. The debridement, antibiotics, irrigation, and implant retention (DAIR) procedure is commonly employed to treat acute, early-stage infections, but its success is highly variable, influenced by factors such as pathogen virulence and antibiotic susceptibility profiles. This study aimed to evaluate the impact of pathogens responsible for these infections on the outcome of DAIR. **Methods**: This retrospective, single-center study analyzed the microbiological profiles of 116 patients (66 hips and 50 knees) treated for acute periprosthetic joint infections (PJIs) with DAIR between 2018 and 2022. Acute PJI was defined as a duration of symptom less than three weeks, according to the criteria established by the Tsukayama and Izakovicova classification. Preoperative joint aspirations, intraoperatively collected tissue samples, and sonication of the exchanged mobile parts were analyzed for each case. We differentiated between monomicrobial PJI, polymicrobial PJI (defined as the identification of more than one microorganism from preoperative joint fluid aspiration or intraoperative samples), and difficult-to-treat (DTT) pathogens. **Results**: In this cohort, the following pathogen profiles were identified: culture-negative cases accounted for 11.1% of infections, while 64.2% were attributed to Gram-positive bacteria, 19.8% to Gram-negative bacteria, and 4.9% to fungal pathogens. Among the identified microorganisms, coagulase-negative staphylococci (CNS) were the most frequently detected, exhibiting a notable oxacillin resistance rate of 52.9% and rifampicin resistance rate of 28.7%. Additionally, no significant difference in revision-free implant survival was found between patients with DTT pathogens and/or polymicrobial PJI and those without such infections. **Conclusions**: This study highlights that pathogens in prosthetic joint infections (PJIs) do not solely determine outcomes, as patient-specific factors (comorbidities, implant type) may also play a key role. Regional variations in pathogens and antibiotic resistance patterns should guide empirical therapy. For instance, this study found a high reliance on vancomycin due to high oxacillin resistance in CNS, the most frequent causative pathogen.

## 1. Introduction

In the field of arthroplasty, periprosthetic joint infection (PJI) remains one of the most devastating complications, contributing to increased morbidity, prolonged hospital stays, and the need for revision surgery. It can significantly reduce the survival rate of the inlying implant. For patients who suffer from an acute infection without implant loosening or signs of a chronic infection, débridement, antibiotics, irrigation, and implant retention (DAIR) is a frequently employed therapy [1,2,3]. The alternative, a two-stage exchange of the prosthesis, is considered the gold standard for treating chronic PJIs. This treatment strategy, however, can pose a significant psychological burden on patients [4]. DAIR offers certain advantages as it enables prosthesis retention when performed successfully. However, the success of DAIR is dependent upon multiple factors, including the causative pathogens. In acute PJI, *Staphylococcus aureus* and coagulase-negative staphylococci (CNS) are the most frequently isolated pathogens [5]. Coagulase-negative staphylococci are opportunistic bacteria form part of the normal skin flora but are also strongly associated with infections related to indwelling medical devices due to their capacity for biofilm formation on prosthetic material [6]. Other bacterial species, such as *Streptococcus* spp. and Enterococcus spp., are less common but can cause acute PJI, often with different resistance profiles and clinical outcomes [7]. Although rare, fungal PJIs (e.g., *Candida* spp.) are described, usually in immunocompromised patients or following multiple prior revisions. These infections are associated with poor outcomes and often necessitate implant removal and subsequent two- or multi-stage exchange [8,9].

Two studies from Wimmer et al. (2016, 2020) have demonstrated that difficult-to-treat (DTT) pathogens and polymicrobial infections can significantly alter the outcome of septic revision arthroplasty [10,11]. Difficult-to-treat organisms pose significant challenges in treatment, as antibiotic options are limited and/or not available in oral form. In this context, rifampicin-resistant staphylococci or ciprofloxacin-resistant Gram-negative bacteria and fungi are commonly classified as DTT pathogens. These patients typically experience longer hospital stays and cannot be treated as effectively as patients with susceptible bacteria. Polymicrobial infections, on the other hand, may require prolonged and more complex antibiotic regimens and may complicate the overall treatment strategy. Furthermore, they are often a sign of more severe infections, typically present in immunocompromised hosts [12].

The microbiological spectrum of acute PJI is diverse. A study by Tai et al. (2022) describes a predominance of Gram-positive organisms, with coagulase-negative staphylococci being the most dominant pathogens, accounting for 37% [13]. In their study, *Staphylococcus aureus* was more prevalent than Gram-negative bacteria. This contrasts with a Spanish multicenter study by Benito et al. (2019), which found that the most common pathogen in acute PJI was *Staphylococcus aureus* [14]. Gram-negative bacteria also contribute significantly to PJI, with *Escherichia coli*, *Proteus mirabilis* and *Pseudomonas aeruginosa* being the most common pathogens in this group [15]. Furthermore, polymicrobial infections occur in both acute and chronic cases, making treatment more difficult [16]. DAIR is performed in cases where, due to an acute onset of symptoms, biofilm has not yet formed and turned sessile. Nevertheless, biofilm-active antibiotics (e.g., rifampicin, fosfomycin) must be administered postoperatively to decrease the probability of biofilm formation and, therefore, treatment failure. Biofilm-producing organisms further complicate infection clearance in patients for whom biofilm-active antibiotics lack oral bioavailability. These patients require targeted antimicrobial therapy based on a comprehensive microbiological assessment. For this, an interdisciplinary approach involving an experienced microbiology department is recommended.

Understanding patients’ microbiological profiles and classifying them in a standardized way could help optimize treatment strategies by harmonizing scientific evidence in this field and, therefore, improving patient outcomes.

This study aims to investigate the microbiological findings in patients with acute early-onset and acute late-onset PJI of the hip and knee who have undergone DAIR, providing insights into pathogen distribution, resistance patterns, and clinical implications for infection management with regard to the inlying implant. Furthermore, this work analyzes the clinical outcome after DAIR as defined by Fillingham et al. (2019) in relation to the pathogens found in microbiology [17]. Another goal of this study is to evaluate the sensitivity and specificity of intraoperative tissue samples compared to preoperative joint aspiration and intraoperative sonication of the exchanged modular parts of the prosthesis.

## 2. Results

A total of 588 samples were taken from 116 infected implants (n = 50 knees (43.1%), n = 66 hips (56.9%)) undergoing DAIR procedure. A pathogen could be detected in 501 samples (85.2%).

From the different tissue samples, joint aspiration, and sonication samples we were able to detect n = 160 different pathogens. Of these, 64.2% were Gram-positive organisms, 19.8% Gram-negative organisms, and 4.9% were yeast.

In the knee subgroup (n = 50), on average, 4.80 ± 1.97 samples were collected from which 2.28 ± 2.54 were positive for microbiology. In the hip subgroup (n = 66), on average, 5.27 ± 1.94 samples were collected from which 2.60 ± 2.50 were positive in microbiology.

We further subdivided the above-mentioned findings into the following categories: coagulase negative staphylococci (n_total = 53; including: n = 24 *Staphylococcus epidermidis*, n = 9 *Staphylococcus haemolyticus*, n = 7 *Staphylococcus capitis*, n = 5 *Staphylococcus lugdunensis*, n = 4 *Staphylococcus hominis*, n = 2 *Staphylococcus warneri*, n = 1 *Staphylococcus lentus*, n = 1 *Staphylococcus caprae*), *Staphylococcus aureus*, *Streptococci*, *Enterococci*, *Proteus mirabilis* and *Escherichia coli*, other Gram-positive pathogens (n = 10 *Cutibacterium acnes*, n = 2 *Cutibacterium avidum*, n = 2 *Corynebacterium amycolatum*, n = 2 *Bacillus cereus*, n = 1 *Finegoldia magna*, n = 1 *Bacillus* spp., n = 1 *Clostridium tertium*, n = 1 *Corynebacterium durum*, n = 1 *Actinomyces*, n = 1 *Pseudarthrobacter sulfivorans*), other Gram-negative pathogens (n = 1 *Proteus vulgaris*, n = 2 *Pseudomonas aeruginosa*, n = 2 *Citrobacter koseri*, n = 1 *Acinetobacter baumannii*, n = 5 *Klebsiella pneumoniae*, n = 1 *Klebsiella oxytoca*, n = 1 *Enterobacter cloacae* complex, n = 1 *Klebsiella aerogenes*, n = 1 *Morganella morganii*), and finally *Fungi* (n_total = 8; n = 8 *Candida* spp.; n = 4, *Candida albicans*, n = 3 *Candida parapsilosis*, n = 1 *Nakaseomyces glabratus*). Table 1 and Table 2 demonstrate the distribution of the pathogens.

To identify potential trends in the pathogen distribution, we deconstructed Table 1 and presented the annual microbiological results in Table 3, covering the years for 2018 to 2022.

**Figure 1:**
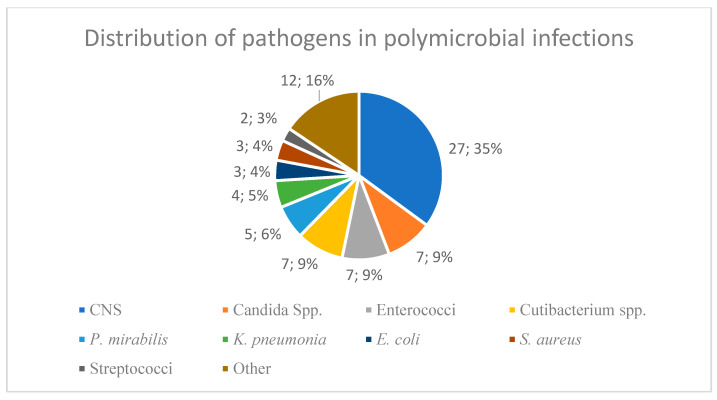
Distribution of pathogens in patients with polymicrobial infection (n = 34). CNS: coagulase-negative staphylococci. Other: n = 2 *Pseudomonas aeruginosa*, n = 1 *Finegoldia magna*, n = 1 *Morganella morganii*, n = 1 *Corynebacterium durum*, n = 1 *Corynebacterium amycolatum*, n = 1 *Clostridium tertium*, n = 1 *Klebsiella oxytoca*, n = 1 *Actinomyces*, n = 1 *Enterobacter cloacae* complex, n = 1 *Acinetobacter baumannii*, n = 1 *Bacillus cereus*.

Following the under material and methods mentioned definition of DTT and polymicrobial infections, we identified a total of 34 cases (30.9%) with DTT pathogens and 31 cases (28.2%) with polymicrobial infections. The highest number of pathogens found in a single polymicrobial patient was four (*Staphylococcus epidermidis*, *Escherichia coli*, *Nakaseomyces glabratus* and *Candida parapsilosis*). This was defined as a polymicrobial infection with DTT due to the presence of a yeast. The different pathogens found in polymicrobial infections are illustrated in Figure 1.

**Figure 2:**
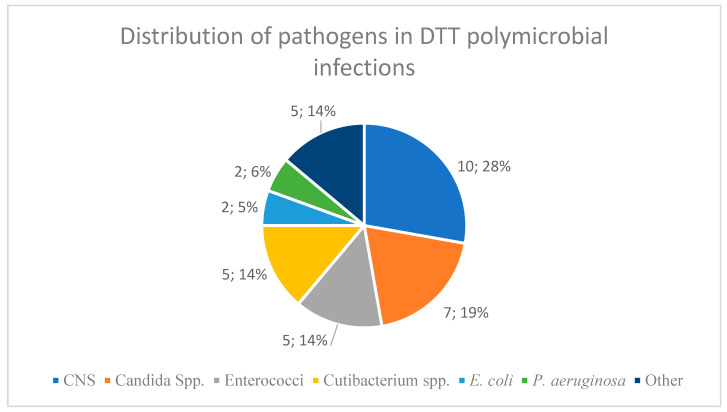
Distribution of pathogens in the n = 15 patients with overlapping DTT polymicrobial infection involving DTT pathogens. CNS: coagulase-negative staphylococci. Other: n = 1 *Enterobacter cloacae* complex, n = 1 *Clostridium tertium*, n = 1 *Acinetobacter baumannii*, n = 1 *Proteus mirabilis*, n = 1 *Klebsiella pneumoniae*.

In a subset of 15 overlapping cases (13.6%), polymicrobial infections involving a DTT pathogen were identified. Figure 2 demonstrates the distribution of the pathogens in these combined cases.

**Figure 3:**
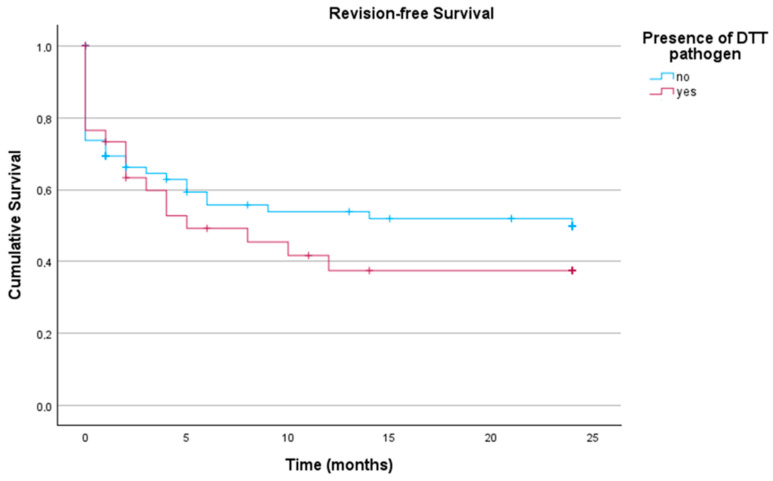
Revision-free implant survival of patients with acute PJI undergoing DAIR with and without DTT pathogens (n_total_ = 116; non-DTT = 82; DDT = 34). DTT: difficult to treat pathogen.

These subgroups were analyzed with regard to revision-free implant survival. As demonstrated in Figure 3, no significant differences in revision-free implant survival outcome between infections caused by non-DTT pathogens and those caused by DTT pathogens (*p* = 0.377) could be detected.

**Figure 4:**
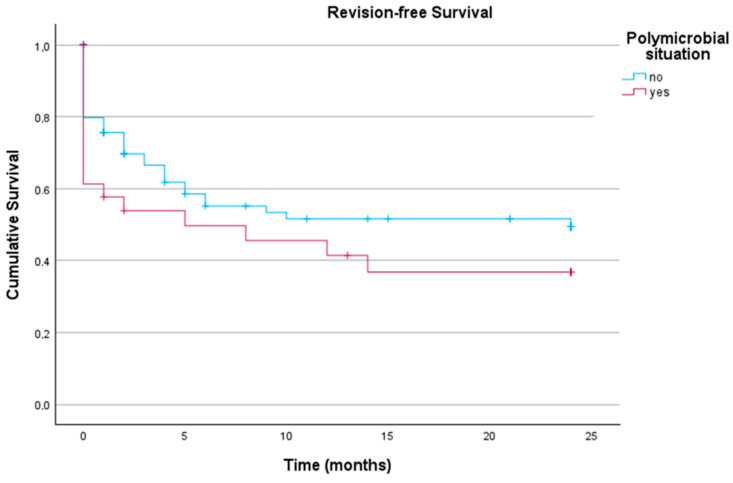
Revision-free implant survival in patients with acute PJI undergoing DAIR comparing cases with and without polymicrobial infections (n_total_ = 116; non-polymicrobial = 85; polymicrobial = 31).

A similar observation was made for patients with polymicrobial versus monomicrobial infections, as these groups showed no significant difference (*p* = 0.170) in the log-rank test of Kaplan–Meier analysis, as demonstrated in Figure 4.

The Kaplan–Meier survival probabilities for the above-mentioned groups is summarized in Table 4.

In the final subgroups analysis, the following groups were compared:

— Neither DTT pathogen nor polymicrobial
— Singular DTT pathogen
— Polymicrobial infection without DTT pathogen
— Polymicrobial infection with DTT pathogen.

**Figure 5:**
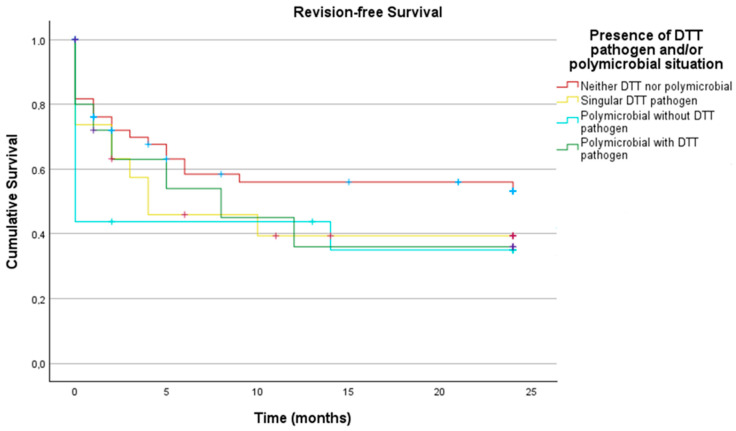
Revision-free survival two years after DAIR in patients: with neither a DTT pathogen nor a polymicrobial infection, with a singular DTT pathogen, with a polymicrobial infection without a DTT pathogen, and with a polymicrobial infection with a DTT pathogen (n_total_ = 116; neither DTT nor polymicrobial = 66; singular-DTT-pathogen = 19, polymicrobial without DTT pathogen = 16; polymicrobial with DTT pathogen = 15).

No statistically significant differences were found between these groups (*p* = 0.329), as shown in Figure 5 with the corresponding Table 5.

For the analysis of antimicrobial susceptibility profiles, we found the following results for *Staphylococcus* spp. as demonstrated in Table 6. Interestingly, among the 25 CNS isolates and two *S. aureus* isolates resistant to Rifampicin, only 13 CNS (52%) were sensitive to fosfomycin as a biofilm-active alternative. Both (100%) rifampicin resistant *S. aureus* found in our collective were susceptible to fosfomycin.

In the *Enterococcus* spp. group, no resistances to ampicillin or vancomycin was detected. For the Gram-negative bacteria, Table 7 demonstrates the resistance rates to piperacillin/tazobactam, ciprofloxacin and meropenem.

## 3. Discussion

The causative pathogens of periprosthetic infections are suspected to significantly influence the treatment outcome of DAIR, single-stage prosthetic exchange, and multi-stage prosthetic exchange. As DAIR remains a viable option for the treatment of acute PJI due to its ability to retain the inlying implant, knowledge about the causative pathogens can help improve treatment algorithms and outcomes. One challenge in managing PJI is associated with culture-negative PJIs (as defined by MSIS), in which the correct antibiotic therapy may not be chosen because of missing microbiological data [18]. In the literature, it is estimated that 5–40% of PJIs are culture-negative and face this problem [19,20,21]. The cohort analyzed in this study had n = 18 (11.1%) culture-negative acute PJIs, which is on the lower end of the reported rates in the literature.

The work published by Kheir et al. (2018) suggests that the optimal number of tissue samples for microbiological analysis during endoprosthetic revision surgery is five [22]. A strength of this study lies in the fact that a total of 588 samples were collected for n = 116 patients, which corresponds to an average of 5.07 (±1.95) samples per patient. Furthermore, the study presented has a homogeneous collective, resulting in n = 66 hips and n = 50 knees, roughly representing the ratio of primary total hip arthroplasties and total knee arthroplasties performed.

To our knowledge, no study to date has reported the yearly distribution of pathogens found in DAIR for acute PJIs. Therefore, comparison with other data is difficult. Across all years analyzed (2018–2022), we were able to show that CNS were the most frequent pathogens detected in microbiology. Moreover, the analysis performed revealed a relatively even distribution of pathogens over the study period, with little temporal fluctuations. The absence of time-dependent abnormalities confirms that the study population is representative, thereby strengthening the validity of the results.

Analogous to the literature, the most common causative pathogen for PJI was CNS in our cohort [13,23]. Some studies suggest that for early infections, typically treated with DAIR, *Staphylococcus aureus* is the most common pathogen [14,15,24]. Our results align with those of Tai et al. (2022), who included n = 2067 PJIs, and found CNS to be the most common pathogen overall. In their acute infection subgroup, however, the relative proportion of *Staphylococcus aureus* was the highest, at 32% [13]. This highlights the need for local microbiological data for endoprothetic referral centers treating PJIs to provide optimal treatment. In comparable studies by Buller et al. (2012) and Tai et al. (2022), Gram-negative pathogens accounted for 11–11.7% in acute infections treated with DAIR [13,15]. Compared to this, our cohort has a relatively high number of Gram-negative pathogens with 19.8%.

Regarding polymicrobial PJIs treated with DAIR, a study by Lora-Tamayo et al. (2013) highlights these infections as a negative factor influencing outcomes following DAIR. In their study, a total of n = 64 (19%) cases were identified with polymicrobial infections, which is relatively low compared to the n = 31 (28.2%) presented in this work [25]. To our knowledge, no comparable studies have been published in the literature. This currently restricts the contextualization and interpretation of the findings.

There are multiple definitions of DTT pathogens in the literature. A frequently used alternative to the definition used in this work is the definition proposed by the PRO-IMPLANT Foundation (version 9, October 2019). According to this definition, DTT pathogens are rifampicin-resistant staphylococci, ciprofloxacin-resistant Gram-negative bacteria and yeasts, as these pathogens tend to form biofilm and, when not susceptible to biofilm-active antibiotics, become difficult to treat [26]. We opted for the definition described by Wimmer and colleagues (2020), as it is a more comprehensive one [11]. We believe that this definition of DTT pathogen goes beyond the PRO-IMPLANT Foundation’s definition, as previous studies (Boisrenoult et al., 2018; da Silva et al., 2021; Henry et al., 2019) have highlighted the challenges of treating *Cutibacterium* spp. (long culture time, relevant biofilm formation), multi-drug-resistant Gram-negative bacteria, and methicillin resistant staphylococci [27,28,29].

Consequently, contemporary PJI classification systems incorporate the causative pathogen as a determinant of revision-free implant survival. For example, the PJI-TNM classification, proposed by Rupp et al. (2021), ranks infections in terms of biofilm formation (acute vs. chronic infections) and the type of pathogen [30]. Here, DTT pathogens are more difficult to treat than non-DTT and culture-negative PJIs, and easier to treat than polymicrobial PJIs and PJIs involving yeasts. Efforts like the PJI-TNM classification are based on the premise that microbiological findings alone cannot determine outcome. In the data presented in this work, polymicrobial and DTT pathogens tend towards worse outcome without reaching statistical significance. With correct surgical treatment, appropriate antibiotic agents, correct dosage, and appropriate treatment duration, this may suggest that, in our cohort, patient and implant-specific factors may have influenced the outcome significantly more than microbiology alone. Nevertheless, it must be considered that a polymicrobial infection or an infection with DTT pathogens can only be detected after microbiological detection of the pathogens. Initial antimicrobial therapy might not be able to cover all possible pathogens.

Empiric antibiotic therapy should be guided by institutional data and continuously optimized through collaboration with the microbiological department. Our data suggest that vancomycin should be used as empirical antibiotic therapy, as n = 46 (52.9%) of the most frequent pathogen (CNS) were resistant to oxacillin. Furthermore, in our cohort resistance to rifampicin of staphylococci could be detected in 23.3% (27 of 116 isolates). Unfortunately, only 52% were sensitive to fosfomycin as a biofilm-active alternative. Representing a relevant proportion. Although biofilm active antibiotics are a cornerstone of antimicrobial therapy, we could not detect an inferior outcome, which is in line with a study by Krizan et al. [31]. Based on the small number of patients receiving a fosfomycin-containing regimen, further studies are necessary. Although the majority of PJI were caused by Gram-positive pathogens, a substantial number of Gram-negative pathogens was observed in this collective (n = 32, 19.8%). Of these, n = 26 (37.7%) were resistant to piperacillin/tazobactam. In critically ill patients with sepsis and an unknown pathogen, adding meropenem to the treatment protocol may be advisable until microbiological culture results are available, as recommended by the Infectious Disease Society of America [32].

### Limitations

The presented study has several limitations. First, the analyzed cohort is derived from an endoprothetic referral center, where patients are transferred in case of complications after surgery or patient-related factors such as multimorbidity. In addition, the retrospective design of the presented study imposes potential selection and documentation bias. Therefore, the microbiological profiles presented may not be directly generalized to all other healthcare settings. Nevertheless, they outline the importance of local epidemiological data for empiric antibiotic therapy. Furthermore, pathogen distribution per year is not sufficient for general recommendation, because of the low number of pathogens per year.

## 4. Materials and Methods

In this retrospective study, we included all consecutive cases of acute PJI of the hip or knee joint treated with DAIR between 2018 and 2022. Acute PJI was defined as a symptom duration of less than three weeks, following the criteria defined by Tsukayama and Izakovicova classifications [26,33]. Exclusion criteria were: radiolucent lines greater than 2 mm as a preoperative sign for implant loosening, a macroscopically loosened implant identified intraoperatively, presence of a sinus tract or fistula, and severe soft tissue impairment which rendered DAIR impossible. For each treated patient, we included the microbiological data from preoperative joint aspiration, intraoperative synovial fluid, and tissue samples, as well as results from sonication of the exchanged mobile components. Sonication was performed in all of the n = 116 cases in this study.

The tissue samples, which were collected intraoperatively, were shredded, homogenized, and then cultured on Columbia agar with 5% sheep blood, MacConkey agar, chocolate agar, and Sabouraud agar (Becton & Dickinson, Bergen County, NJ, USA). This was also performed with 0.5 mL of sonication fluid. Additionally, 1 mL of the sample was transferred into thioglycolate broth (Becton & Dickinson, Bergen County, NJ, USA). Schaedler and kanamycin/vancomycin agar plates (Becton & Dickinson, Bergen County, NJ, USA) were used for anaerobic cultures. These were streaked with shredded and homogenized intraoperative tissue samples. For the evaluation of the sonication fluid, 0.5 mL of sonication fluid was streaked on culture plates. The incubation conditions of the cultures were 5% CO_2_ at 35 °C for a minimum of 14 days. In addition, to the analysis of culture growth, the sonication fluid was added to PEDS medium blood culture flasks (Becton & Dickinson, Bergen County, NJ, USA) and incubated for 14 days in a Bactec FX blood culture system (Becton & Dickinson, Bergen County, NJ, USA). Similarly, the preoperative joint aspiration fluid was inoculated into PEDS medium blood culture flasks (Becton & Dickinson, Bergen County, NJ, USA) and incubated for 14 days in a Bactec FX blood culture system (Becton & Dickinson, Bergen County, NJ, USA). The microbiological procedures were performed according to the methods described by Fröschen et al. (2022) [34].

Demographic data such as body mass index (BMI), age, and the number of prior surgeries were collected [35]. We differentiated between monomicrobial PJI, polymicrobiological PJI (identification of more than one distinct microorganism from preoperative joint fluid aspiration or intraoperative samples) and difficult-to-treat (DTT) PJI as previously described by Wimmer et al. (2020), which was based on a classification from Zimmerli et al. (2004) [11,36]. In brief, the following pathogens were defined as DTT:

— Methicillin-resistant *Staphylococcus aureus*
— Methicillin-resistant coagulase-negative staphylococci
— Rifampicin or Fluorquinolone resistant staphylococci
— *Staphylococcus* spp. resistant to doxycycline, linezolid, and trimethoprim/sulfamethoxazole (insufficient oral bioavailability)
— *Enterococci* resistant to Ampicillin
— Gram-negative bacteria with resistances to Meropenem, Piperacillin/Tazobactam or Fluorquinolone
— Extended-spectrum β-lactamases (ESBL) and AmpC-producing Enterobacterales
— *Cutibacterium* (*Propionibacterium*) spp.
— Fungi

The antibiotic susceptibility profiles of the pathogens and the infection-free postoperative follow-up after initial treatment were analyzed. For each patient, we included (when available) one antibiotic susceptibility profile from sonication fluid, the tissue sample and preoperative joint aspiration, as these are the microbiological samples on which treatment decisions were based. The definition of a successful outcome was defined as revision-free survival of more than two years, as also defined by Fillingham et al. (2019) [17].

All patients with acute PJIs and well-fixed implants underwent a standardized treatment protocol, which included 2 weeks of targeted intravenous (i.v.) antibiotic therapy followed by four weeks of oral antibiotic therapy after initial DAIR surgery. After identification of the causative pathogen, antibiotic therapy was adapted according to the antibiotic susceptibility profile. We did not perform continuous suppression therapy in patients with acute infections. Our first-line targeted intravenous therapy was the combination of vancomycin with rifampicin. The administration of fosfomycin was generally limited to cases where rifampicin resistance was present, or administration of rifampicin was not possible (e.g., comorbidities). In patients with no oral antibiotic therapy available, i.v. antibiotic therapy was continued for a total of 6 weeks. Clinical and radiological follow-ups were conducted at six weeks, six months, and twelve months, with yearly follow-ups thereafter. Particular attention during follow-up was paid to local signs of persistent infection, or implant loosening, defined as progressive radiolucent lines or migration of the inlying components.

Routine blood analyses for inflammatory markers were carried out, and in cases of suspected recurrent infection, joint aspiration was performed to analyze the fluid for total cell count and microbiological growth (culture and polymerase chain reaction (PCR)).

### Statistical Analysis

Microsoft Excel 2024 (Microsoft Corporation, Richmond, VA, USA) was used for data compilation, while statistical analyses were performed using SPSS Statistics version 28 for Windows (SPSS, Inc., an IBM company, Chicago, IL, USA). Descriptive statistics, including arithmetic means, standard deviations, and ranges, were calculated. Unless stated otherwise, results are expressed as means ± standard deviation (SD).

A Kaplan–Meier analysis was performed to evaluate revision-free implant survival based on the pathogen found in microbiological testing. To compare the Kaplan–Meier analysis of different subgroups (DTT, non-DTT, monomicrobial, polymicrobial, neither DTT nor polymicrobial) to each other a log-rank test was used (*p* < 0.05).

## 5. Conclusions

The pathogens detected in PJI do not appear to solely determine clinical outcomes, emphasizing the importance of patient-specific factors (e.g., comorbidities, type of inlying implant). Although no significant differences were detected, identifying DTT pathogens remains important, as their presence can guide the clinician in tailoring antibiotic therapy for optimal infection control. Institutional variability in pathogen distribution and antibiotic susceptibility profiles underscores the importance of maintaining local antibiogram data to guide empirical therapy—an often-underestimated factor in many published studies on PJI treatment outcomes. At our institution, for example, vancomycin is the first-line agent for coagulase-negative staphylococci (CNS) due to limited alternatives and high oxacillin resistance rates. Institution-specific pathogen profiles should be analyzed locally, and vancomycin should be considered an empiric antibiotic agent against acute periprosthetic joint infections.

## Figures and Tables

**Table 1:** Overall distribution of pathogens identified in DAIR revision arthroplasty for acute periprosthetic infection.

Type of Pathogen	N	Percentage %
sterile	18	11.1
Gram-positive	104	64.2
Gram-negative	32	19.8
Fungi	8	4.9
Total	162	100.0

**Table 2:** Distribution of pathogens identified in DAIR revision arthroplasty for acute periprosthetic infection.

Type of Pathogen	N	Percentage %
Sterile	18	11.1
Gram-positive bacteria		
Coagulase-negative *Staphylococci*	53	33.1
*Staphylococcus aureus*	12	6.9
*Streptococci*	10	6.3
*Enterococci*	7	4.4
Other Gram-positive	22	13.7
Gram-negative bacteria		
*E. coli*	9	5.6
*P. mirabilis*	8	4.4
Other Gram-negative	15	9.3
Fungi	8	5.0
Total	162	100

**Table 3:** Yearly distribution of pathogens identified via microbiological diagnostics in 116 patients with acute PJI treated with DAIR from 2018 and 2022. Data based on unique samples from sonication, intraoperative tissue samples or preoperative joint fluid aspiration. Abbreviations: CNS: coagulase-negative staphylococci. DAIR: débridement, antibiotics, irrigation and implant retention. Other Gram-positive: see above.

Year	2018	2019	2020	2021	2022
DAIR per year	12	30	26	26	22
CNS	4 (26.6%)	13 (32.5%)	17 (53.1%)	11 (32.4%)	8 (34.8%)
*Staphylococcus aureus*	1 (6.7%)	3 (7.5%)	3 (9.4%)	2 (5.9%)	3 (13.1%)
*Streptococci*	1 (6.7%)	3 (7.5%)	1 (3.1%)	4 (11.8%)	1 (4.3%)
*Enterococci*	3 (20.0%)	2 (5.0%)	0 (0%)	2 (5.9%)	0 (0%)
Other Gram-positive	2 (13.3%)	10 (25.0%)	4 (12.5%)	3 (8.8%)	3 (13.0%)
*E. coli*/*Proteus mirabilis*	1 (6.7%)	6 (15.0%)	2 (6.3%)	3 (8.8%)	5 (21.7%)
Other Gram-negative	2 (13.3%)	2 (5.0%)	5 (15.6%)	3 (8.8%)	3 (13.1%)
*Fungi*	1 (6.7%)	1 (2.5%)	0 (0%)	6 (17.6%)	0 (0%)
Total pathogens	15	40	32	34	23

**Table 4:** Revision-free implant survival in patients with acute PJI undergoing DAIR, stratified by the presence or absence of DTT pathogens and/or polymicrobial infections. DTT: difficult to treat pathogen.

Time in Months	6	12	24
Non-DTT (n = 82)	55.7%	53.9%	49.8%
DTT (n = 34)	49.2%	37.5%	37%
Non-polymicrobial (n = 85)	55.2%	51.6%	49.5%
Polymicrobial (n = 31)	49.7%	41.4%	36.8%
Polymicrobial and DTT (n = 15)	54%	36%	36%

**Table 5:** Revision-free survival two years after DAIR in patients: with neither a DTT pathogen nor a polymicrobial infection, with a singular DTT pathogen, with a polymicrobial infection but without a DTT pathogen, and with a polymicrobial infection with DTT pathogen.

Revision-Free Survival (Months)	6	12	24
Neither (n = 66)	58.4%	56%	53.2%
Singular DTT (n = 19)	45.9%	39.4%	39%
Polymicrobial without DTT (n = 16)	43.8%	43%	35%
Polymicrobial with DTT (n = 15)	54%	36%	36%

**Table 6:** Resistance of Coagulase-negative staphylococci and *Staphylococcus aureus* to oxacillin, rifampicin and vancomycin.

		Coagulase-Negative Staphylococci	*Staphylococcus aureus*	Total
Oxacillin	Resistant	46	0	46
	Susceptible	41	29	68
Rifampicin	Resistant	25	2	27
	Susceptible	62	27	89
Vancomycin	Resistant	1	0	1
	Susceptible	86	29	115
Total		87	29	116

**Table 7:** Resistance profiles of Gram-negative bacteria to piperacillin/tazobactam, ciprofloxacin and meropenem.

		Gram-Negative Bacteria	Total
Piperacillin/Tazobacatam	Resistant	26	69
	Susceptible	43	
Ciprofloxacin	Resistant	7	69
	Susceptible	62	
Meropenem	Resistant	0	69
	Susceptible	69	

## Data Availability

The original contributions presented in this study are included in the article; further inquiries can be directed to the corresponding author.
